# Analysis of multidrug resistant group B streptococci with reduced penicillin susceptibility forming small, less hemolytic colonies

**DOI:** 10.1371/journal.pone.0183453

**Published:** 2017-08-17

**Authors:** Hirotsugu Banno, Kouji Kimura, Yosuke Tanaka, Tsuyoshi Sekizuka, Makoto Kuroda, Wanchun Jin, Jun-ichi Wachino, Keiko Yamada, Keigo Shibayama, Yoshichika Arakawa

**Affiliations:** 1 Department of Bacteriology, Nagoya University Graduate School of Medicine, Showa-ku, Nagoya, Aichi, Japan; 2 Department of Microbiology Laboratory, Yokohama City Seibu Hospital, St. Marianna University School of Medicine, Asahi-ku, Yokohama, Kanagawa, Japan; 3 Pathogen Genomic Center, National Institute of Infectious Diseases, Shinjyuku-ku, Tokyo, Japan; 4 Department of Bacteriology II, National Institute of Infectious Diseases, Musashi-Murayama, Tokyo, Japan; The University of Hong Kong, HONG KONG

## Abstract

Group B streptococci (GBS; *Streptococcus agalactiae*) are the leading cause of neonatal invasive diseases and are also important pathogens for elderly adults. Until now, nearly all GBS with reduced penicillin susceptibility (PRGBS) have shown β-hemolytic activity and grow on sheep blood agar. However, we have previously reported three PRGBS clinical isolates harboring a CylK deletion that form small less hemolytic colonies. In this study, we examined the causes of small, less hemolytic colony formation in these clinical isolates. Isogenic strains were sequenced to identify the mutation related to a small colony size. We identified a 276_277*ins*G nucleic acid insertion in the thiamin pyrophosphokinase (*tpk*) gene, resulting in premature termination at amino acid 103 in TPK, as a candidate mutation responsible for small colony formation. The recombinant strain Δ*tpk*, which harbored the 276_277*ins*G insertion in the *tpk* gene, showed small colony formation. The recombinant strain Δ*cylK*, which harbored the G379T substitution in *cylK*, showed a reduction in hemolytic activity. The phenotypes of both recombinant strains were complemented by the expression of intact TPK or CylK, respectively. Moreover, the use of Rapid ID 32 API and VITEK MS to identify strains as GBS was evaluated clinical isolates and recombinant strains. VITEK MS, but not Rapid ID 32 API, was able to accurately identify the strains as GBS. In conclusion, we determined that mutations in *tpk* and *cylK* caused small colonies and reduced hemolytic activity, respectively, and characterized the clinical isolates in detail.

## Introduction

*Streptococcus agalactiae* (GBS) is the leading cause of neonatal sepsis and meningitis and is responsible for high mortality and morbidity, particularly in neonates and those suffering from underlying medical conditions, such as diabetes [1–3]. β-Lactams are first-line antimicrobial agents for intrapartum antibiotic prophylaxis and the treatment of GBS infections [4, 5]. However, GBS clinical isolates with reduced penicillin susceptibility (PRGBS) have emerged via the acquisition of substitutions, including V405A and/or Q557E, in penicillin-binding protein 2X [6–10]. PRGBS clinical isolates tend to be non-susceptible or resistant to fluoroquinolones and macrolides [11–13]. Most PRGBS clinical isolates show β-hemolytic activity and grow on sheep blood agar. However, we previously reported three multidrug-resistant PRGBS clinical isolates (MRY11-004, MRY11-005, and NUBL-2449) that form atypical, small less-β-hemolytic colonies on sheep blood agar [14]. These clinical isolates harbor a G379T nucleic acid substitution in the *cylK* gene, resulting in premature termination at amino acid 127 in CylK, which is required for full hemolytic activity of GBS[14].

Small colony variants (SCVs) are characterized by reduced growth, small colony size, and atypical colony morphology. Additional features, such as decreased respiration, increased resistance to aminoglycosides, reduced fermentation of sugars, and an unstable phenotype are common. SCVs are often linked to a deficiency in electron transport or thymidine biosynthesis [15], but the precise causes of small colony formation in other cases are unclear [16–20]. Although studies of the morphological and biochemical characteristics of SCVs have been most extensively studied in staphylococci [21–23], SCVs are found in various genera and species, e.g., enterococci [17, 18, 24], *Streptococcus pneumoniae* [19, 20], *Streptococcus tigurinus* [16], *Escherichia coli* [25], and *Pseudomonas aeruginosa* [15].

To our knowledge, there is one report of GBS opacity variants obtained from an unknown number of passages of a clinical isolate [26]. However, there are no previous reports of clinical GBS small colony variants. In this report, we elucidated the causes of reduced hemolytic activity and small colony formation in three clinical isolates of PRGBS.

## Materials and methods

### Bacterial strains and culture conditions

The three clinical isolates (MRY11-004, MRY11-005, and NUBL2449) were recovered from two patients in one hospital in 2011. The first patient was an 88-year-old man who had underlying diseases, including diabetic peripheral neuropathy. The isolates were recovered in January of 2011. MRY11-004 and MRY11-005 were isolated from his blood and sputum, respectively. The second patient was an 83-year-old man. NUBL-2449 was isolated from his sputum in November of 2011. All three clinical isolates were classified as ST1. Moreover, all three clinical isolates showed an identical pulsotype according to PFGE. The details of these clinical isolates are described in [14].

The GBS type V strain ATCC BAA-611 (2603 V/R) and type Ia ATCC BAA-1138 (A909) were used as the parent strains to generate recombinants. The GBS recombinant strain Δ*cylK* was based on ATCC BAA-611, harboring the G379T substitution in *cylK*, resulting in premature termination at amino acid 127 in CylK. The GBS recombinant strain Δ*tpk* was based on ATCC BAA-1138, harboring the 276_277*ins*G insertion in *tpk*, resulting in premature termination at amino acid 103 in thiamin pyrophosphokinase (TPK), which catalyzes the direct phosphorylation of thiamin via ATP to form thiamin pyrophosphate (TPP).

GBS was cultivated in Todd–Hewitt broth (THB) (BD, Franklin Lakes, NJ, USA) and Todd–Hewitt agar (THA) at 37°C in 5% CO_2_. GBS strains carrying recombinant pG+host6 or pDL278 derivatives were grown in the presence of erythromycin (5 μg/ml), chloramphenicol (10 μg/ml), and/or spectinomycin (300 μg/ml). Cultivation of Δ*tpk* was performed at 37°C in 5% CO_2_ in THB and on THA containing TPP (500 μg/L). *E*. *coli* DH10B was grown at 37°C in LB broth and strains carrying recombinant pG+host6 or pDL278 derivatives were selected in the presence of ampicillin (200 μg/ml) and spectinomycin (200 μg/ml).

### Next-generation sequencing and analysis

Bacteria were cultured overnight at 37°C in 5% CO_2_, and chromosomal DNA was extracted using the QIAamp^®^ DNA Mini Kit (Qiagen, Hilden, Germany) following the manufacturer’s protocol, with slight modifications in order to completely remove RNA. The overnight culture (1 mL) was centrifuged for 10 min at 3,000 × *g*, and the supernatant was removed. The pellet was resuspended in 200 μL of the suspension solution and then incubated at 37°C for 90 min. Then, 20 μL of proteinase K was added, and the solution was incubated for 60 min at 57°C, until it became clear. RNase A (4 μL) was added, and the solution was incubated for 5 min at 25°C. DNA was then purified following the QIAamp DNA Kit protocol. Concentrations of extracted DNA were measured using both NanoDrop and QuBit fluorometric quantitation. Extracted DNA was then prepared for next-generation sequencing using the Nextera^®^ XT DNA Preparation Kit. Sequencing was performed using a MiSeq sequencer (Illumina, San Diego, CA, USA) with the MiSeq Reagent Kit v3 (2 × 300 mer). Paired-end sequencing data from the MiSeq reporter software were further analyzed using CLC genomics Workbench ver. 9 (CLC Bio).

### Construction of Δ*cylK* and a complementation plasmid to express intact CylK in Δ*cylK*

pG+host6-Δ*cylK* was constructed to introduce the G379T substitution into *cylK*. The plasmid was comprised of a thermosensitive plasmid pG+host6 backbone, with a fragment of the *cylK* gene containing the G379T substitution (nucleotide positions 659180 to 659657 in the GBS ATCC BAA-611 genome), the chloramphenicol acetyltransferase gene, conferring resistance to chloramphenicol, and a fragment of DNA from the region directly downstream of the *cylK* gene (nucleotide positions 659073 to 659648 in the GBS ATCC BAA-611 genome). These fragments were amplified by PCR using primers listed in S1 Table. Then, pG+host6-Δ*cylK* was transformed into *E*. *coli* DH10B for amplification. The purified plasmid was transformed into GBS BAA-611 and the transformants were selected on THA containing 0.5 μg/ml erythromycin at 30°C. Successful integrant strains were then cultivated for 3 days in THB at 30°C without erythromycin selection to facilitate the excision of the vector pG+host6-Δ*cylK* [27].

The pCylK plasmid was constructed to express full-length CylK in Δ*cylK*. pCylK was comprised of the *E*. *coli*-GBS shuttle vector plasmid pDL278 backbone, with a fragment of the promoter region from the *bca* gene (nucleotide positions 459015 to 459255 in the GBS ATCC BAA-1138 genome) and the full-length *cylK* gene (nucleotide positions 459015 to 459255 in the GBS ATCC BAA-611 genome) [28, 29]. The pCylK plasmid was transformed into *E*. *coli* DH10B for amplification. The purified plasmid was transformed into Δ*cylK* and the transformants were selected on THA containing 300 μg/ml spectinomycin.

### Construction of Δ*tpk* and a complementation plasmid to express intact TPK in Δ*tpk*

Δ*tpk* was constructed using a similar method to that used for the construction of Δ*cylK*, with minor modifications. pG+host6-Δ*tpk* was constructed in order to introduce the 276_277*ins*G insertion in *tpk*. The plasmid was comprised of a thermosensitive plasmid pG+host6 backbone, with a fragment of the *tpk* gene containing the 276_277*ins*G insertion (nucleotide positions 1790161 to 1790826 in the GBS ATCC BAA-1138 genome), the chloramphenicol acetyltransferase gene, and a fragment of DNA from the region directly downstream of the *tpk* gene (nucleotide positions 1789494 to 1790190 in the GBS ATCC BAA-1138 genome). Integrant strains were successively cultivated for 3 days in THB containing TPP (500 μg/L) at 30°C without erythromycin selection to facilitate the excision of vector pG+host6-Δ*tpk*.

The pTPK plasmid was constructed to express full-length TPK in Δ*tpk*. pTPK was comprised of the *E*. *coli*-GBS shuttle vector plasmid pDL278 backbone, with a fragment of the promoter region from the *bca* gene (nucleotide positions 459015 to 459255 in the GBS ATCC BAA-1138 genome), and the full-length *tpk* gene (nucleotide positions 1790174 to 1790806 in the GBS ATCC BAA-1138 genome). pTPK was transformed into *E*. *coli* DH10B for amplification. The purified plasmid was transformed into Δ*tpk* and MRY11-004. Thereafter, transformants were selected on THA containing 300 μg/ml spectinomycin at 37°C.

### Growth curve

Bacterial growth in THB at 37°C in ambient air was monitored using an OD monitor (ODBox-C; TAITEC, Koshigaya, Japan). Overnight culture (60 μL) was diluted in 6 mL of fresh THB. The cultures were shaken at 160 rpm and the optical density (600 nm) was measured every 5 min for 24 h. The experiment was performed five times.

### Transmission electron microscopy

For transmission electron microscopy (TEM) analyses, BAA-1138 and Δ*tpk* cultures were incubated for 12 h in THB and THB containing TPP (500 μg/L), respectively. MYR11-004 and Δ*tpk* cultures were also incubated for 12 h in THB containing no TPP. After washing with PBS (0.1 M Phosphate Buffer Solution, pH 7.4) three times, bacterial cells were washed in PBS containing 2% glutaraldehyde and then stored at 4°C overnight. Bacterial cell pellets were washed with PBS four times, resuspended in PBS containing 2% osmium tetroxide, and incubated at 4°C for 2 h. Samples were cut using an ultramicrotome. TEM was performed using the JEM-1400PLUS (JEOL, Tokyo, Japan).

### Rapid ID 32 API and VITEK MS assay

Clinical isolates, Δ*tpk*, and BAA-1138 were analyzed using Rapid ID 32 API (bioMérieux, Marcy-l'Étoile, France) and VITEK MS (Sysmex bioMérieux) according to the manufacturer’s instructions. The three clinical isolates and Δ*tpk* were cultured on sheep blood agar (Nissui, Tokyo, Japan) for 3 days and on chocolate II agar (BD) in 5% CO_2_ overnight. BAA-1138 cultured on sheep blood agar and on chocolate II agar in 5% CO_2_ overnight was also analyzed.

### Nucleotide sequence accession numbers

Raw sequence data from Miseq sequencing are deposited as fastq files at EMBL/GenBank under the accession numbers SAMD00077745, SAMD00077746, SAMD00077747, SAMD00077757, SAMD00077758 and SAMD00077759 for MRY11-004, MRY11-005, NUBL-2449, MRY11-004L, MRY11-005L and NUBL-2449L, respectively. The raw sequencing data have been deposited in the DDBJ Sequence Read Archive (DRA) under accession numbers DRA005682, DRA005683, DRA005684, DRA005686, DRA005687, and DRA005688 for MRY11-004, MRY11-005, NUBL-2449, MRY11-004L, MRY11-005L, and NUBL-2449L, respectively [30].

### Statistical analysis

Growth curve data are expressed as means ± standard deviation. The statistical significance (*P*-values) of differences in mean values for two-sample comparisons was determined using the Student's *t*-test implemented in Microsoft Excel. *P* < 0.05 indicated statistical significance [31].

## Results

### Phenotypes of the Δ*cylK* strain

**Fig 1:**
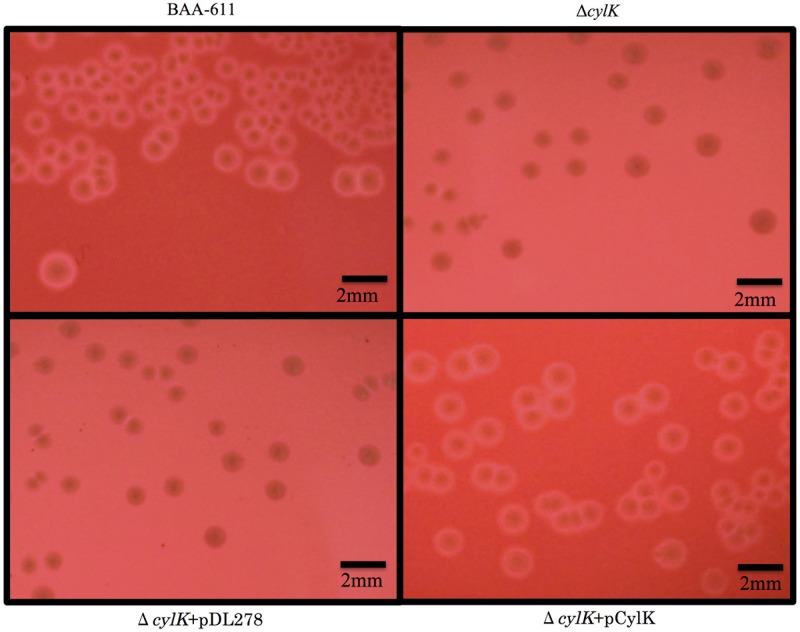
Comparison of hemolytic activity and colony formation to elucidate influence of deletion CylK. Colony size and hemolytic activity of a GBS ATCC strain (BAA-611), recombinant strains (Δ*cylK*), and complemented strains (Δ*cylK*+ pDL278 and Δ*cylK*+pCylK) on 5% sheep blood agar. The strains were grown at 37°C in 5% CO_2_ for 16 h. Δ*cylK* is a recombinant strain based on ATCC BAA-611, harboring the G379T substitution in *cylK*, resulting in premature termination at amino acid 127 in CylK. pDL278 is a gram-positive and gram-negative shuttle vector. pCylK is a complementation plasmid used to express intact TPK in the Δ*cylK* strain.

To determine if Δ*cylK* causes reduced hemolytic activity and/or small colony formation, we constructed Δ*cylK* based on BAA-611 harboring the G379T substitution in *cylK* gene. The recombinant strain showed less β-hemolytic activity than that of the wild-type strain on sheep blood agar plates. However, Δ*cylK* did not show small colony formation (Fig 1). The mutant was then complemented with a plasmid harboring *cylK* and the resulting strain showed similar hemolytic activity to that of the wild type on sheep blood agar plates (S1 Fig).

### Identification of the cause of small colony formation and phenotypes of the Δ*tpk* strain

**Fig 2:**
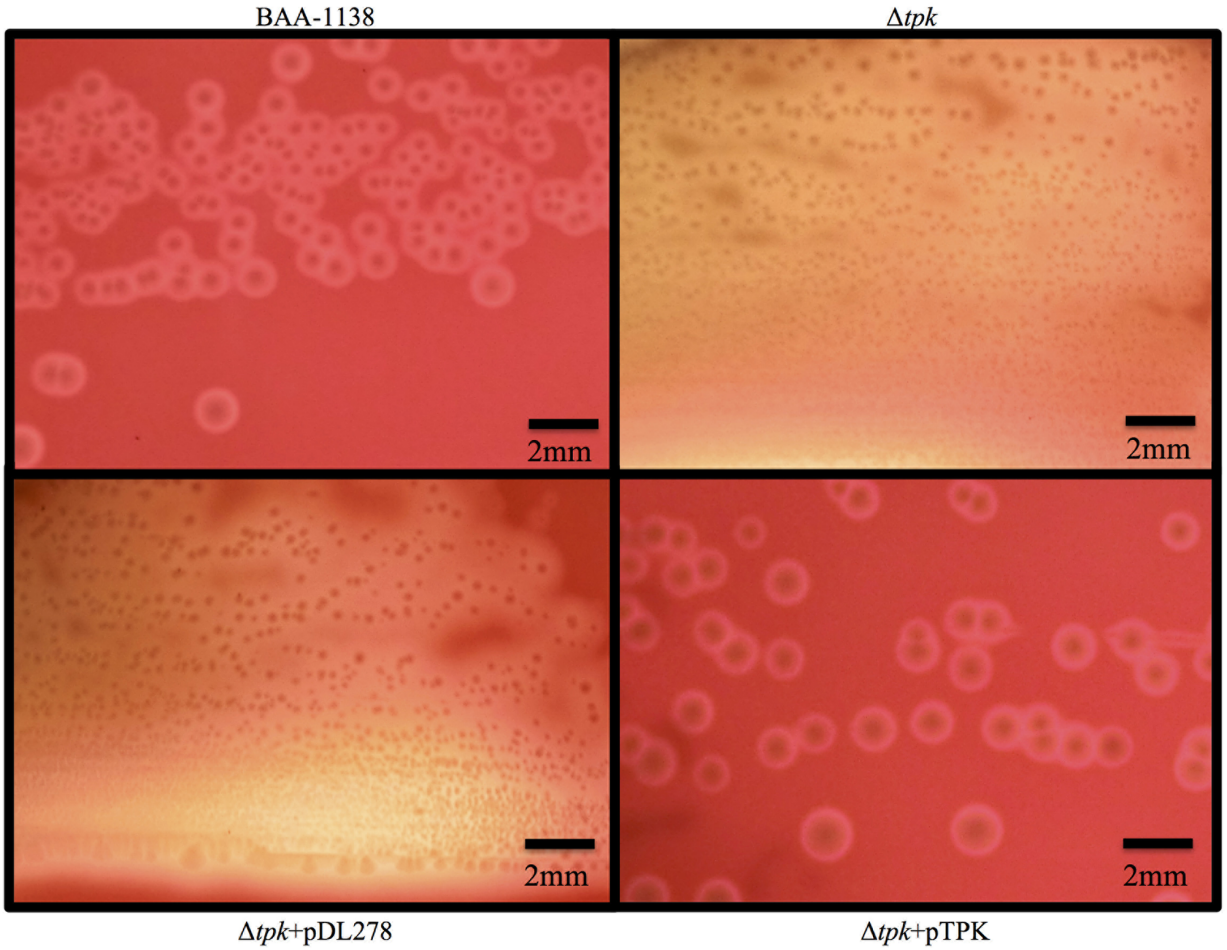
Comparison of hemolytic activity and colony formation to elucidate influence of deletion TPK. Colony morphology of BAA-1138, Δ*tpk*, Δ*tpk*+pDL278, and Δ*tpk*+pTPK grown on sheep blood agar. BAA-1138 and Δ*tpk*+pTPK were grown at 37°C in 5% CO_2_ for 16 h. Δ*tpk* and Δ*tpk*+pDL278 were grown at 37°C in 5% CO_2_ for 72 h. Δ*tpk* is a recombinant strain based on ATCC BAA-1138, harboring the 276_277*ins*G insertion in *tpk*, resulting in a deletion in premature termination at amino acid 103 in thiamin pyrophosphokinase (TPK). pDL278 is a gram-positive and gram-negative shuttle vector. pTPK is a complementation plasmid used to express intact TPK in the Δ*tpk* strain.

After several *in vitro* passages on 5% sheep blood agar, three clinical isolates reverted to a fast-growing phenotype and large colony size, equal to that of the GBS ATCC strains (approximately 1 mm in diameter). Large colonies were confirmed to be GBS using the agglutination method with anti-Lancefield B antigen serum and serotype VIII with anti-GBS serotype-specific serum, and these results were identical to those obtained for clinical isolates. To identify a causal genetic factor for the observed differences, the whole genomes of the clinical isolates and the derivative strains were sequenced. Genomes were compared using CLC Workbench ver. 9 to detect single nucleotide polymorphisms (SNPs). Although several SNPs were found in comparisons between the clinical isolates and derivative strains (Table 1), only the 276_277*ins*G insertion in the *tpk* gene was found in all three clinical isolates. This insertion was confirmed using Sanger sequencing, and caused premature termination at amino acid 103 in TPK. To determine the effects of this insertion, we constructed the Δ*tpk* recombinant strain based on *S*. *agalactiae* ATCC BAA-1138 harboring the 276_277*ins*G insertion in the *tpk* gene. The Δ*tpk* strain showed similar hemolytic activity on sheep blood agar to that of the wild-type strain (S1 Fig) and smaller colonies (less than 1 mm in diameter) than those of the wild-type strain (approximately 1 mm in diameter) on sheep blood agar (Fig 2). Moreover, when a plasmid expressing full-length TPK was introduced into Δ*tpk* and MRY11-004, the resulting strain showed similar colony formation to that of the wild-type strain on sheep blood agar (Fig 2).

**Table 1:** Nucleic acid differences between MRY11-004 and MRY11-004L, MRY11-005 and MRY11-005L, NUBL-2449 and NUBL-2449L using CLC workbench ver. 9 (>80%; Frequency).

Nucleic acid difference		Position	Effect (length with the insertion or deletion/full length of the gene)	Gene description (Locus tag)
MRY11-004	MRY11-004L			
G	-	276_277	Frameshift (102/210)	thiamine pyrophosphokinase (SAG1775)
A	G	78 bases upstream of SAG2072 (78 bases upstream of SAG2073)	-	uridine phosphorylase (SAG2072) (GntR family transcriptional regulator (SAG2073))
-	C	226_227	Frameshift (80/515)	Phosphoribosyl aminoimidazolecarboxamide formyltransferase/IMP cyclohydrolase (SAG0030)
-	C	341_342	Frameshift (115/1275)	Mannosyl-glycoprotein endo-β-*N*-acetylglucosaminidase (EN73_03515)
MRY11-005	MRY11-005L			
G	-	276_277	Frameshift (102/210)	thiamine pyrophosphokinase (SAG1775)
NUBL2449	NUBL2449L			
G	G	276_277	Frameshift (102/210)	thiamine pyrophosphokinase (SAG1775)
C	-	279		thiamine pyrophosphokinase (SAG1775)
-	C	1442_1443	Frameshift (501/692)	elongation factor G (SAG1769)

The strains MRY11-004L, MRY11-005L, and NUBL2449L are large colony strains derived from their parental clinical small colony strains MRY11-004, MRY11-005, and NUBL2449, respectively, after several passages on sheep blood agar.–indicates a deletion. Position is defined as the distance from the start codon. Frequency = Count (detected specific nucleic acids at a nucleic acid position)/Coverage (all nucleic acids at a certain nucleic acid position).

### Comparative analysis of growth characteristics

**Fig 3:**
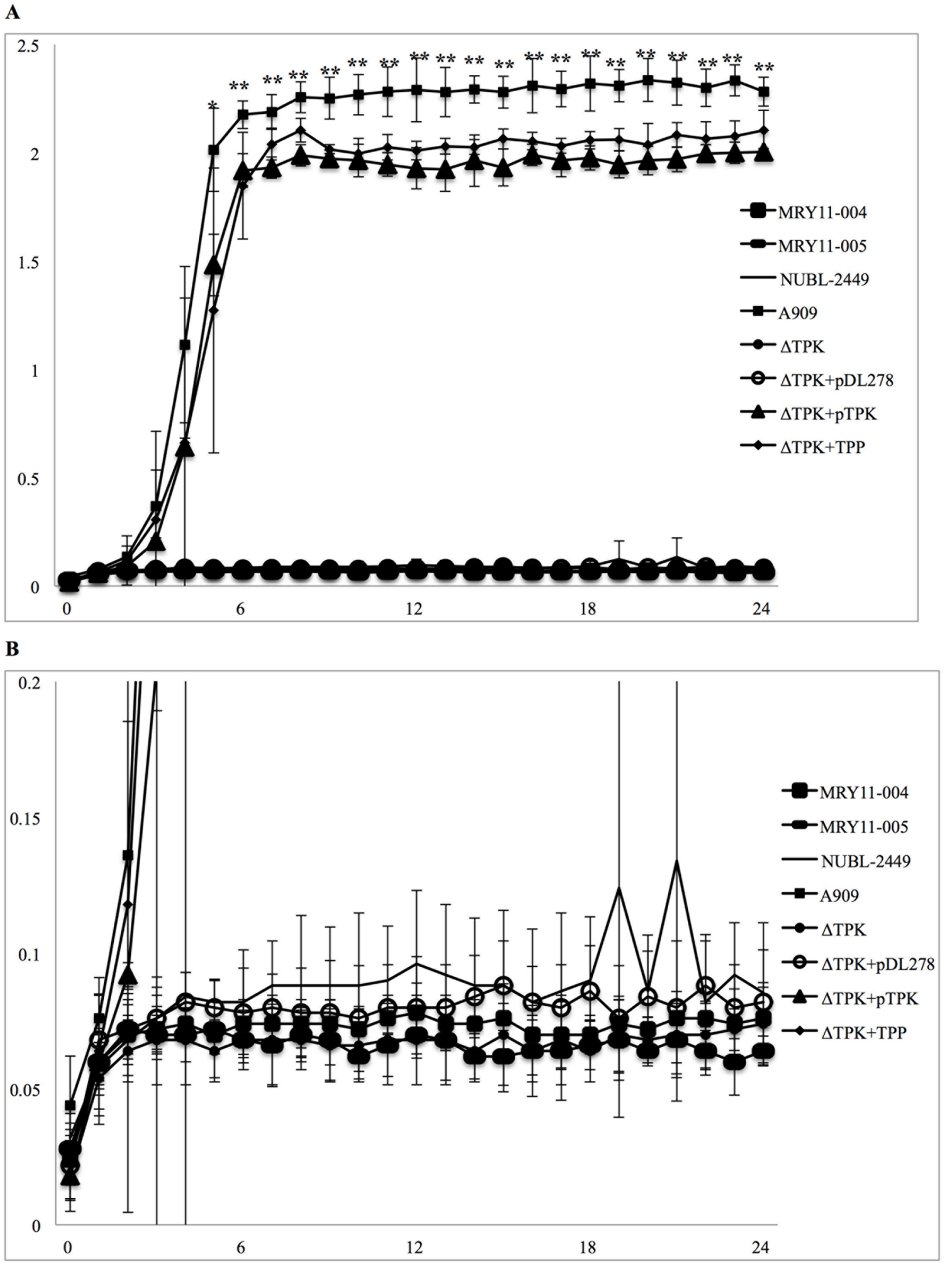
Comparison of growth rate of GBS ATCC strain, recombinant strain and complemented strains. Growth curves (OD_600nm_) of the clinical isolates (MRY11-004, MRY11-005, and NUBL-2449), GBS ATCC strain (BAA-1138), recombinant strain (Δ*tpk*), and complemented strains (Δ*tpk*+pDL278 and Δ*tpk*+pTPK) at 37°C in ambient air (A,B). OD_600nm_ = 0–2.5 (A), OD_600nm_ = 0–0.2 (B). Data are presented as averages of five independent experiments. Data are the mean ± standard deviation OD_600nm_. *,**Statistically significant difference: *P < 0.05; **P < 0.01. Δ*tpk* indicates a recombinant strain based on ATCC BAA-1138, harboring the 276_277*ins*G insertion in *tpk*, resulting in premature termination at amino acid 103 in thiamin pyrophosphokinase (TPK). pDL278 is a gram-positive and gram-negative shuttle vector. pTPK is a complementation plasmid used to express intact TPK in the Δ*tpk* strain. Δ*tpk*+TPP indicates Δ*tpk* grown in Todd–Hewitt broth containing thiamine pyrophosphate (500 μg/L).

We monitored the growth of the clinical isolates GBS ATCC strain BAA-1138, Δ*tpk*, Δ*tpk*+pDL278, and Δ*tpk*+pTPK. The GBS ATCC BAA-1138, Δ*tpk*+pTPK, and Δ*tpk* supplemented with TPP (500 μg/L) had similar growth profiles. However, the clinical isolates Δ*tpk* and Δ*tpk*+pDL278 displayed significantly reduced growth compared with that of the GBS ATCC strain BAA-1138, Δ*tpk* supplemented with TPP (500 μg/L), and Δ*tpk*+pTPK (Fig 3).

### Auxotrophic testing

**Fig 4:**
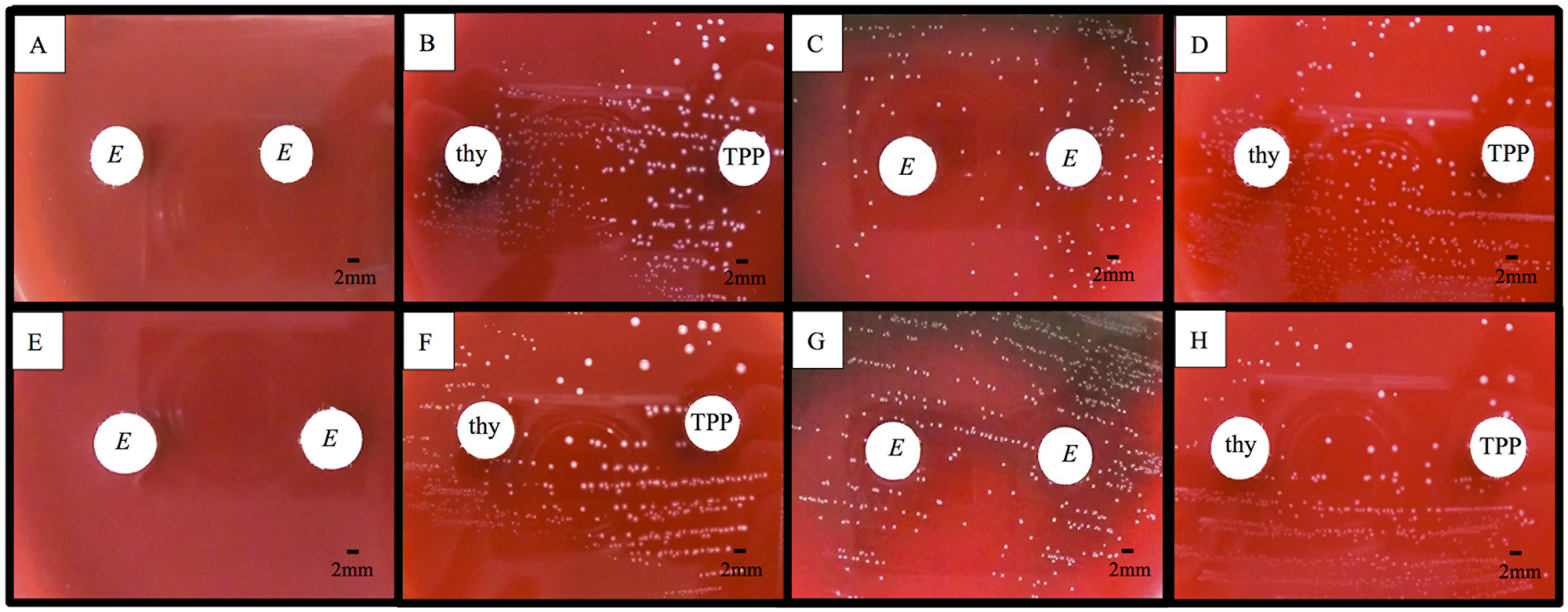
Comparison of auxotrophy of GBS ATCC strain, recombinant strain and complemented strains. Colony morphology of the clinical isolate MRY11-004 (A–D) and recombinant strain Δ*tpk* (E–H) grown on sheep blood agar plate (A, B, E, F) and Mueller Hinton Agar with 5% sheep blood (C, D, G, H) after incubation in 5% CO_2_ overnight. In B, D, F, and H, the left disc contained 20 μg of thymidine (thy) and the right disc contained 20 μg of thiamin pyrophosphate (TPP). In A, C, E, and G, both the left and right discs were empty (*E*). Δ*tpk* is a recombinant strain based on ATCC BAA-1138, harboring the 276_277*ins*G insertion in the thiamin pyrophosphokinase (*tpk*) gene, resulting in premature termination at amino acid 103 in TPK.

The clinical isolates and Δ*tpk* showed similar colony sizes to those of GBS ATCC strains on sheep blood agar, MHA, and THA containing greater than 50 μg/L TPP. Additionally, similar to A909, these isolates grew on sheep blood agar around discs containing 20 μg of TPP. However, on MHA with 5% sheep blood and sheep blood agar around discs with 20 μg of thymidine, Δ*tpk* showed medium colony sizes (1 mm in diameter), which were smaller than those of GBS ATCC strains (Fig 4). Moreover, THA containing more than 50 μg/ml thymidine showed medium-sized colonies. Although previous reports have shown that the addition of NAD^+^, menadione, thiamine, or hemin results in a normal colony morphology in *S*. *aureus* SCVs, the clinical isolates and Δ*tpk* did not show visible growth around discs containing 20 μg of these compounds[15] [21] [22].

While the clinical isolates and Δ*tpk* were able to grow on chocolate II agar, they showed medium colony sizes on BY chocolate agar. A comparison of the ingredients in these agars revealed that chocolate II agar contained 10 mg/L hemoglobin, while BY chocolate agar contained 5% horse blood. Accordingly, the hemoglobin content may explain the growth of the clinical isolates and Δ*tpk*. Supplementation of MHA and THA with 10 mg/mL hemoglobin confirmed this hypothesis; the clinical isolates and Δ*tpk* showed colony sizes similar to those of the ATCC strain.

### TEM analyses

**Fig 5:**
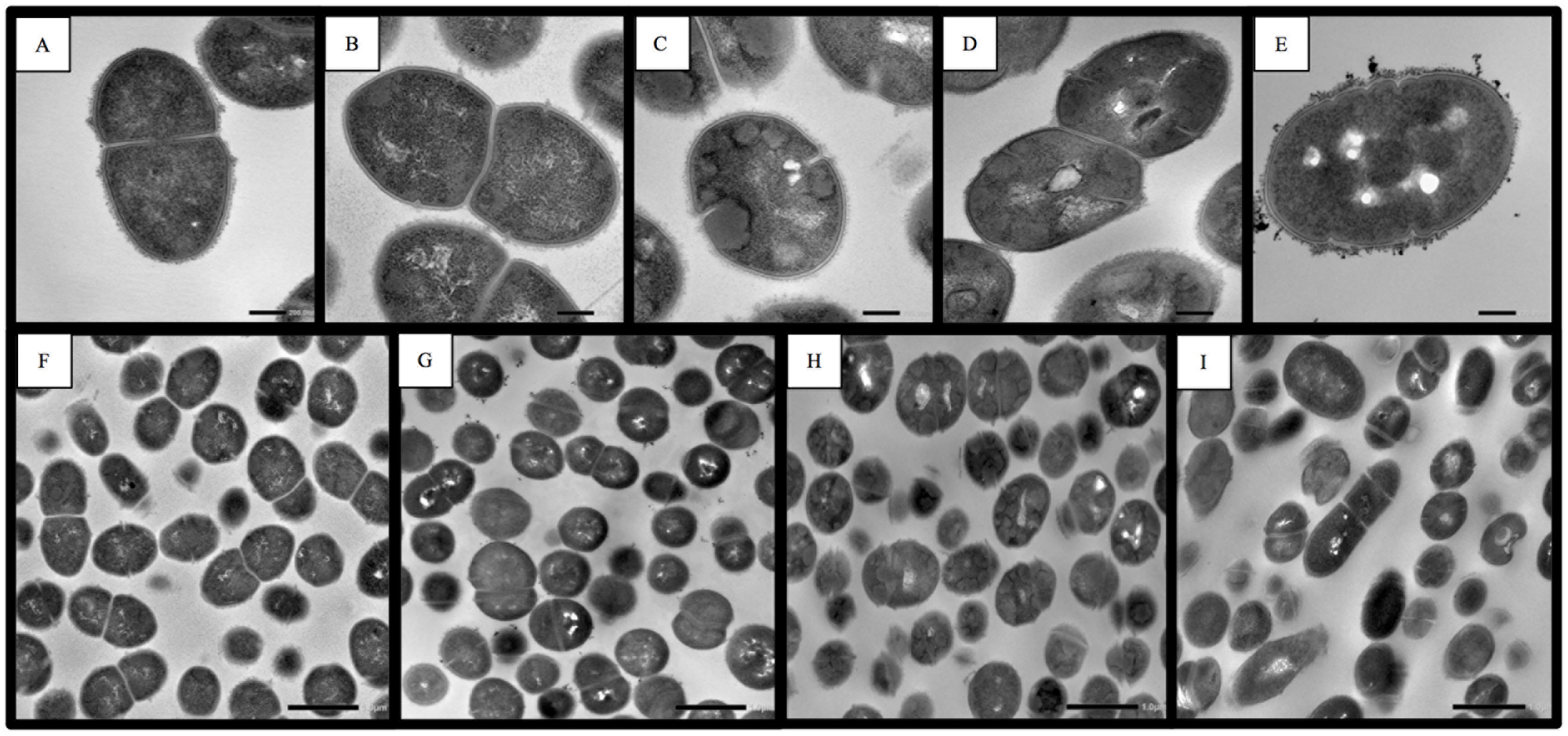
Comparison of TEM analysis of GBS ATCC strain, recombinant strain and complemented strains. TEM of BAA-1138 (A and F), Δ*tpk* with TPP 500 μg/L (B and G), Δ*tpk* (C and H), and MRY11-004 (D, E and I). Bars: 200 nm (A–E). Bars: 1 μm (F–I) Δ*tpk* is a recombinant strain based on ATCC BAA-1138, harboring the 276_277*ins*G insertion in *tpk*, resulting in premature termination at amino acid 103 in thiamin pyrophosphokinase.

BAA-1138 and Δ*tpk* containing TPP (500 μg/L) exhibited regular cell division with single cross walls in dividing cells. However, cell division in MRY11-004 and Δ*tpk* appeared to terminate before the cells could fully separate and show agglutination. Moreover, only MRY11-004 showed heterogeneous cell sizes and cell clusters (Fig 5).

### Identification by Rapid ID 32 Strep API and VITEK MS

Although the clinical isolates and BAA-1138 were identified as GBS using the Rapid ID 32 Strep API system, Δ*tpk* cultured on sheep blood was not identified as GBS and was judged as SDSE or GBS. Concerning biological profiles, the clinical isolates showed different trehalose (TRE), pullulan (PUL), sodium pyruvate (VP), and maltose (MAL) phenotypes compared to those of BAA-1138. Moreover, in addition to PUL, VP, and MAL, Δ*tpk* showed different sucrose (SAC) phenotypes. Therefore, Δ*tpk* was unable to be correctly confirmed as GBS. However, the clinical isolates, Δ*tpk*, and BAA-1138 cultured on sheep blood and chocolate II agar were identified as GBS (99.9% identity) using VITEK MS.

## Discussion

We characterized the first clinical PRGBS with less hemolytic activity and auxotrophy for TPP. The G379T substitution in *cylK* gene and 276_277*ins*G insertion in *tpk* gene caused the less hemolytic activity and small colony formation, respectively. In previous reports, SCVs with electron transport and thymidine biosynthesis defects have shown both small colony formation and low hemolytic activity due to the deletion of a single gene. However, in this study, small colony formation and reduced hemolytic activity were caused by different genes. The deletion of CylK results in reduced hemolytic activity on horse blood agar and similar growth to that of the parental strain in THB supplemented with 5% yeast extract [32, 33]. This suggests that full-length CylK is required for full hemolytic activity of GBS, and may not be related to cell growth and colony size. The G379T substitution in *cylK*, resulting in premature termination at amino acid 127 in CylK, was associated with low hemolytic activity. The deletion of amino acids 22 to 115 of CylK have been reported to cause reduced hemolytic activity [32]. Therefore, full-length CylK might be required for full hemolytic activity in GBS. In the TEM analysis, a clinical isolate showed heterogeneous cell sizes and cell clusters; however, there are many candidate loci associated with this heterogeneity based on next-generation sequence data for the three clinical isolates and therefore the underlying causes are unknown.

The Δ*tpk* strain, which exhibited premature termination at amino acid 103 in TPK, showed small colony formation. Moreover, complementation of the strain with a plasmid harboring full-length TPK resulted in similar colony sizes to those of GBS ATCC strains. Therefore, the function of this domain is related to growth. The structure of TPK in yeast bound with TPP has revealed the locations of the thiamin-binding site and probable catalytic residue [34]. Analysis of GBS TPK using Pfam showed that these sites were conserved. Therefore, because amino acid positions 103–210 in TPK correspond to the thiamine-binding domain, Δ*tpk* and clinical isolates lacking these amino acids might not bind thiamine and accordingly might be unable to catalyze the production of TPP. Moreover, because TPP, a coenzyme form of vitamin B1, is important for the formation of a coenzyme required for central metabolic functions (pyruvate decarboxylase, pyruvate dehydrogenase, α-ketoglutarate dehydrogenase, and transketolase), TPP might be essential for cell growth in bacteria and Δ*tpk* might exhibit growth defects [35]. Moreover, in *Schizosaccharomyces pombe*, *tnr3*-recombinant strains (low-expressed TPK) grow slowly and show aberrant morphology [36]. The TPK deletion has not been detected in bacterial clinical isolates; therefore, these are the first known clinical isolates with the 276_277*ins*G insertion in the *tpk* gene.

The three clinical isolates analyzed in this study somewhat resemble previously reported SCVs. However, they also have different characteristics from previously reported SCVs. The three clinical isolates showed slow growth phenotypes on THA, sheep blood agar, and in THB. This phenotype resembles that of previously reported SCVs [15–18, 24]. However, the clinical isolates in this study did not show irregular cell shapes, as observed in other SCVs, when analyzed by TEM [17, 18], nor did they show auxotrophy for menadione, hemine, or thiamine, as did SCVs of *S*. *aureus*. The clinical isolates and Δ*tpk* showed auxotrophy for TPP. Moreover, small colony formation was caused by a partial deletion in the TPK, which has not been reported in other SCVs to date. In general, electron transport deficient-SCVs and thymidine biosynthesis deficient-SCVs show an increase in gentamicin and sulfamethoxazole/trimethoprim MIC, respectively. Although the three clinical isolates showed resistance to sulfamethoxazole/trimethoprim (MIC, >256 μg/ml), there was no difference observed in the MIC of gentamicin for the clinical isolate MRY11-004 (MIC 32 μg/ml) compared to those of MRY11-004+pTPK (MIC 32 μg/ml, expressing full-length TPK in MRY11-004), Δ*tpk* (MIC 64 μg/ml), and Δ*tpk* +pTPK (MIC 64 μg/ml). Furthermore, SCVs are typically isolated from patients undergoing long-term antibiotic therapy, and can cause latent or recurrent infections [15]. For the three clinical isolates, sulbactam/ampicillin therapy was documented from October 25 to October 31 and from November 28 to December 2 in 2010. Thereafter, two of the clinical isolates (MRY11-004 and MRY11-005) were isolated from sputum and blood on January 4, 2011. Moreover, piperacillin/tazobactam and levofloxacin therapy was documented from October 22 to October 25 and from November 27 to November 31 in 2010, respectively. The clinical isolate NUBL-2449 was isolated from sputum on November 14, 2012 [14]. Therefore, no cases of long-term antibiotic therapy were documented in the two patients, and this likely does not explain the small colony formation phenotype. On the other hand, these β-lactam and quinolone therapies might have selected for multidrug resistance and/or PRGBS. Selection for PRGBS by long-term β-lactam therapy has been observed [9]. Additionally, conventional identification systems (e.g., the API Rapid system) have failed to identify *E*. *coli*, *E*. *faecium*, and *E*. *faecalis* SCVs correctly [18, 24, 25]. Although the clinical isolates cultured on chocolate II agar and sheep blood agar could be detected using the Rapid ID 32 Strep API system, Δ*tpk* on sheep blood agar yielded no definitive results owing to an inconsistent biological phenotype. Furthermore, several biochemical phenotypes were different from those of the parental strain and GBS ATCC strains. Therefore, clinical isolates of GBS with deletions in the *tpk* gene might be misidentified in the future. In contrast to conventional identification systems based on biochemical characteristics, the clinical isolates and Δ*tpk* were correctly identified by VITEK MS (99.9% identity). Moreover, *E*. *faecium* SCVs were precisely identified by VITEK MS [18]. These findings suggest that MALDI-TOF MS is a powerful tool for the identification of strains with similar atypical phenotypes.

In conclusion, we characterized the first PRGBS clinical isolates with reduced hemolytic activity and auxotrophy for TPP. Moreover, we found that VITEK MS correctly identified GBS with a deletion in TPK. In this study, we revealed the causes of small colony formation with less hemolytic activity and characterized clinical isolates in detail. Because these clinical isolates may be misclassified using routine bacterial tests and are multidrug resistant, they represent a potential public health concern.

## Supporting information

S1 Fig Comparison of hemolytic activity of clinical isolates, recombinant strains and complemented strains. Clinical isolates(MRY11-004, MRY11-005 and NUBL-2449), recombinant strains(Δ*cylK* and Δ*tpk*) and complemented strains (Δ*cylK*+pDL278, Δ*cylK*+pCylK, Δ*tpk*+pTPK and Δ*tpk*+pTPK) on Mueller Hinton Agar with 5% Sheep Blood. Δ*cylK* indicates a recombinant strain based on ATCC BAA-611, harbouring the G379T substitution in *cylK*, resulting in premature termination at amino acid 127 in CylK. pDL278 is a Gram-positive and gram-negative shuttle vector. Δ*cylK*+pCylK indicates a complementation plasmid to express intact TPK in Δ*cylK* strain.Δ*tpk* indicates a recombinant strain based on ATCC BAA-1138, harbouring the 276_277*ins*G insertion in *tpk*, resulting in premature termination at amino acid 103 in thiamin pyrophosphokinase. Δ*tpk*+pTPK indicates a complementation plasmid to express intact TPK in Δ*tpk* strain. A: MRY11-004 B: MRY11-005 C: NUBL-2449 D: 2603 V/R E; Δ*cylK* F: Δ*cylK* +pDL278 G: Δ*cylK*+CylK H: Δ*cylK*+pCylK I: A909 J; Δ*tpk* K: Δ*tpk*+pDL278 L; Δ*tpk*+pTPK.
(TIFF)

S1 Table Primers used for PCR amplification in this study. (DOCX)
