# Supplementary figures and images for: Analysis of multidrug resistant group B streptococci with reduced penicillin susceptibility forming small, less hemolytic colonies
Source: PLoS One. 2017 Aug 17;12(8):e0183453. doi: 10.1371/journal.pone.0183453 (PMC5560676; doi:10.1371/journal.pone.0183453)

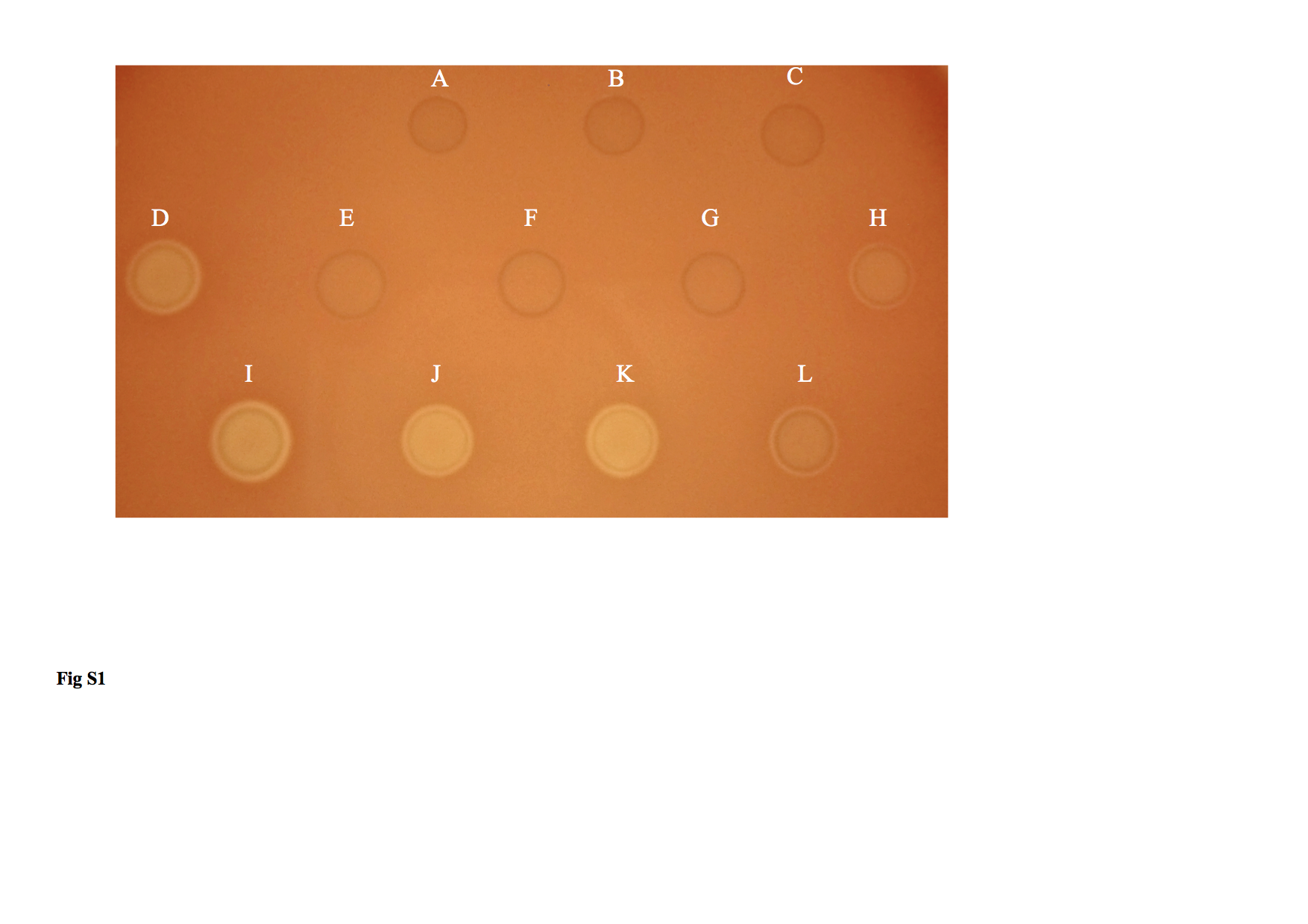

Supplement: S1 Fig — Clinical isolates(MRY11-004, MRY11-005 and NUBL-2449), recombinant strains(ΔcylK and Δtpk) and complemented strains (ΔcylK+pDL278, ΔcylK+pCylK, Δtpk+pTPK and Δtpk+pTPK) on Mueller Hinton Agar with 5% Sheep Blood. ΔcylK indicates a recombinant strain based on ATCC BAA-611, harbouring the G379T substitution in cylK, resulting in premature termination at amino acid 127 in CylK. pDL278 is a Gram-positive and gram-negative shuttle vector. ΔcylK+pCylK indicates a complementation plasmid to express intact TPK in ΔcylK strain.Δtpk indicates a recombinant strain based on ATCC BAA-1138, harbouring the 276_277insG insertion in tpk, resulting in premature termination at amino acid 103 in thiamin pyrophosphokinase. Δtpk+pTPK indicates a complementation plasmid to express intact TPK in Δtpk strain. A: MRY11-004 B: MRY11-005 C: NUBL-2449 D: 2603 V/R E; ΔcylK F: ΔcylK +pDL278 G: ΔcylK+CylK H: ΔcylK+pCylK I: A909 J; Δtpk K: Δtpk+pDL278 L; Δtpk+pTPK. (TIFF) [file pone.0183453.s001.tiff]
