# Supplementary material for: Analysis of multidrug resistant group B streptococci with reduced penicillin susceptibility forming small, less hemolytic colonies
Source: PLoS One. 2017 Aug 17;12(8):e0183453. doi: 10.1371/journal.pone.0183453 (PMC5560676; doi:10.1371/journal.pone.0183453)
Supplement: S1 Table — (DOCX) [file pone.0183453.s002.docx]

**S1 Table**

| Primer Name | Primer (5′–3′) |
| --- | --- |
| CylK_up_f_SalI | GACGTCGACACAAGCAAATAGACAGGTAAGCA |
| CylK_up_r | TGTTGCGAGTTAGTTATCTGGT |
| Chloramphenicol_f | TGGCAACGTGAATTTAGA |
| Chloramphenicol_r_BamHⅠ | GGTGGATCCGTATATTCTAGCCACTTC |
| CylK-down_f_BamHⅠ | GGTGGATCCCGTTAGCTGGTTAACAAACCG |
| CylK-_down_r_XbaI | GGATCTAGAATAGGCTGCGGCACACTTTA |
| Promoter_f_HindIII | GCGAAGCTTGAGTTACCACCTTTGATTTAATATGTT |
| Promoter_r_SphI | GCGGCATGCAATATTCTCCTTTACTCTGTATAACTCTATAC |
| TPK_up_f_BamHI | GAGAGGATCCAACCTTAAGGGTGGCGCTC |
| TPK_up_r_EcoRI | GACTGAATTCGATGACAAGC ATATTAACCT CTATCC |
| Chloramphenicol_f_EcoRI | GACTGAATTCTGGCAACGTGAATTTAGA |
| Chloramphenicol_r_HindIII | GACTAAGCTTGTATATTCTA GCCACTTC |
| TPK_down_f_HindIII | GACTAAGCTTGTAAGGATAGAGGTTAATATGCTTGTC |
| TPK_down_r_KpnI | GACTGGTACCCTCCCTGACCATTTCCAGGT |
| CylK-full_down_f_SphI | CGCGCATGCTTTGAATCCTTTATCAGAAAAAGAC |
| CylK-full_down_r_EcoRI | CGCGAATTCTTAGTTATCTGGTAATAATTCTTTTAAGTAAAC |
| TPK-full_down_f_SphI | GCAGGCATGCATGACTAAAATTGCTTTATTTGCTGG |
| TPK-full_down_r_EcoRI | GGCAGAATTCGACAAGCATATTAACCTCTATCCTTAC |
